# Epigallocatechin-3-Gallate (EGCG) Promotes Autophagy-Dependent Survival via Influencing the Balance of mTOR-AMPK Pathways upon Endoplasmic Reticulum Stress

**DOI:** 10.1155/2018/6721530

**Published:** 2018-01-31

**Authors:** Marianna Holczer, Boglárka Besze, Veronika Zámbó, Miklós Csala, Gábor Bánhegyi, Orsolya Kapuy

**Affiliations:** ^1^Department of Medical Chemistry, Molecular Biology and Pathobiochemistry, Semmelweis University, Budapest, Hungary; ^2^Pathobiochemistry Research Group of the Hungarian Academy of Sciences and Semmelweis University, Budapest, Hungary

## Abstract

The maintenance of cellular homeostasis is largely dependent on the ability of cells to give an adequate response to various internal and external stimuli. We have recently proposed that the life-and-death decision in endoplasmic reticulum (ER) stress response is defined by a crosstalk between autophagy, apoptosis, and mTOR-AMPK pathways, where the transient switch from autophagy-dependent survival to apoptotic cell death is controlled by GADD34. The aim of the present study was to investigate the role of epigallocatechin-3-gallate (EGCG), the major polyphenol of green tea, in promoting autophagy-dependent survival and to verify the key role in connecting GADD34 with mTOR-AMPK pathways upon prolonged ER stress. Our findings, obtained by using HEK293T cells, revealed that EGCG treatment is able to extend cell viability by inducing autophagy. We confirmed that EGCG-induced autophagy is mTOR-dependent and PKA-independent; furthermore, it also required ULK1. We show that pretreatment of cells with EGCG diminishes the negative effect of GADD34 inhibition (by guanabenz or siGADD34 treatment) on autophagy. EGCG was able to delay apoptotic cell death by upregulating autophagy-dependent survival even in the absence of GADD34. Our data suggest a novel role for EGCG in promoting cell survival via shifting the balance of mTOR-AMPK pathways in ER stress.

## 1. Introduction

Green tea is a type of traditional Chinese tea made from *Camellia sinensis* leaves, and it has been demonstrated to possess profound biochemical and pharmacological activities, including antioxidative, anti-inflammatory, and anticarcinogenic properties [1–3]. Green tea contains several polyphenolic components, for example, catechin, epicatechin, and epigallocatechin-3-gallate (EGCG). Effects of the most abundant green tea polyphenol EGCG have been shown in various pathophysiological conditions, including insulin resistance, endothelial dysfunction, and ischemia-reperfusion injuries [4–6]. Many scientific reports proposed that green tea is able to influence several biological processes by inhibiting telomerase, mitogen-activated protein kinase (MAPK), activator protein-1, or nuclear factor- (NF-) *κ*B [7]. It has been also shown that EGCG is able to extend longevity significantly under several stress conditions by postponing aging and age-related diseases [8–10].

Cellular homeostasis is finely controlled by an evolutionarily conserved cytoprotective cellular digestive process, called autophagy [11]. Cells have residual autophagic activity even under physiological conditions; however, the process gets more efficient during various stress events (i.e., starvation and growth factor deprivation) [12]. During autophagy, cellular components become sequestered into a double-membrane vesicle, whose contents are then delivered to and degraded by lysosomes [13, 14]. Due to the crucial role of autophagy in maintaining cellular homeostasis, this self-eating process is precisely regulated [11]. Interestingly, an excessive level of autophagy is also known to cause cell death [15].

One of the key roles of autophagy is to maintain essential cellular activity and viability under limited nutrient availability [13]. Therefore, autophagy is tightly controlled by the two sensors of nutritional conditions, called mTOR and AMPK [16–18]. mTOR (mammalian target of rapamycin) is a serine/threonine protein kinase in the mTORC1 complex, which is the main component of the mTOR pathway [18, 19]. This complex is a master regulator by integrating inputs from external and internal signals, such as growth factors, amino acids, glucose, and energy status, to control growth and metabolism [20]. Besides mTOR, AMPK (AMP-activated protein kinase) also senses cellular energy status and has a crucial role in maintaining energy homeostasis [21]. AMPK tightly controls ATP-consuming processes, such as glycogen or protein syntheses, and it upregulates processes that yield ATP (i.e., glycolysis) [22, 23]. AMPK is able to promote self-eating by phosphorylating ULK1, one of the main inducers of autophagosome formation [17, 21]. However, ULK1 is also regulated by an mTOR-dependent inhibitory phosphorylation under nutrient-rich condition [21]. In addition, AMPK directly inhibits mTORC1 complex via phosphorylation [17, 21] indicating that a proper balance of AMPK-mTOR pathways is essential at physiological conditions.

It has been lately suggested that EGCG might induce a cytoprotective autophagy in various stress events. Treatment with EGCG promotes the formation of autophagosomes both in primary bovine endothelial and human hepatoma (HepG2) cells [24, 25]. EGCG abolishes the palmitate-induced accumulation of lipid droplets via facilitated autophagic flux [24]. Autophagy enhancement upon EGCG administration is unfavourable for hepatitis B virus replication, and hence it is considered as a potential therapeutic strategy [25]. EGCG also has neuroprotective effect by activating autophagy and inhibiting Bax and cytochrome *c* translocation in prion-protein-induced damages [26]. Although the positive role of EGCG in enhancing autophagy at various diseases has been already suggested, details of the regulatory mechanisms induced by this natural compound are yet to be revealed.

Huang et al. have suggested that EGCG upregulates AMPK activity in a dose-dependent manner, while mTOR pathway gets inhibited in hepatoma cells [27]. A docking experiment has also shown that EGCG is an ATP-competitive inhibitor of mTOR [28]. Kim et al. suggested that EGCG enhances autophagy through an AMPK-mediated mechanism [24]. Interestingly, EGCG stimulated both AMPK and ULK1, but not mTOR, indicating that the polyphenol-induced autophagy is independent from mTOR pathway [24]. These results suggest that EGCG acts as an enhancer on AMPK; however, its effect on mTOR pathway is still contradictory.

Recently, we have confirmed that activation of autophagy has a cytoprotective role upon high level of endoplasmic reticulum (ER) stress [29, 30]. This transient elevation of autophagy is characterized by downregulation of mTOR and upregulation of AMPK. Therefore, mTOR inhibitors and/or AMPK activators (such as rapamycin, resveratrol, and metyrapone) are able to postpone apoptotic cell death during excessive ER stress [29, 31]. EGCG is able to restore Ca^2+^ homeostasis suggesting its cytoprotective effect in ER stress [32]; however, the detailed mechanism of the EGCG-modulated ER stress response remains to be elucidated.

In this study, we investigate the mechanism of EGCG-dependent autophagy and its role in ER stress by using a human cell line. We propose that the cytoprotective autophagy stimulated by EGCG is regulated via both mTOR and ULK1. We also show that EGCG-induced self-eating process is independent from PKA. Here, we present that EGCG affects the balance of mTOR-AMPK, which delays apoptotic cell death by upregulating autophagy upon ER stress. Our data demonstrate a novel mechanism underlying the effect of EGCG on life-and-death decision in ER stress.

## 2. Materials and Methods

### 2.1. Materials

Thapsigargin (Sigma-Aldrich, T9033), tunicamycin (Sigma-Aldrich, T7765), rapamycin (Sigma-Aldrich, R0395), guanabenz (Sigma-Aldrich, G110), H89 (Adipogen, AG-CR1-0002), and epigallocatechin gallate (Sigma-Aldrich, E4143) were purchased. All other chemicals were of reagent grade.

### 2.2. Cell Culture and Maintenance

A human embryonic kidney cell line (HEK293T, ATCC, and CRL-3216) was used as a model system. It was maintained in DMEM (Life Technologies, 41965039) medium supplemented with 10% fetal bovine serum (Life Technologies, 10500064) and 1% antibiotics/antimycotics (Life Technologies, 15240062). Culture dishes and cell treatment plates were kept in a humidified incubator at 37°C in 95% air and 5% CO_2_.

### 2.3. SDS-PAGE and Western Blot Analysis

Cells were harvested and lysed with 20 mM Tris, 135 mM NaCl, 10% glycerol, and 1% NP40, pH 6.8. Protein content of cell lysates was measured using Pierce BCA Protein Assay (Thermo Scientific, 23225), and equal amounts of proteins were used in the analysis. SDS-PAGE was done by using Hoefer miniVE (Amersham). Proteins were transferred onto Millipore 0.45 *μ*M PVDF membrane. Immunoblotting was performed using TBS Tween (0.1%), containing 5% nonfat dry milk, 1% bovine serum albumin (Sigma-Aldrich, A9647), or gelatin buffer (Sigma-Aldrich, G8327) for blocking membrane and for antibody solutions. Loading was controlled by developing membranes for GAPDH or by dying them with Ponceau S in all experiments. At least three independent measurements were carried out in each experiment. The following antibodies were applied: antiLC3B (SantaCruz, sc-16755), antiCaspase3 (SantaCruz, sc-7272), antiPARP (Cell Signaling, 9542S), antiULK-555-P (Cell Signaling, 5869S), antiULK (Cell Signaling, 8054S), antip70S6-P (Cell Signaling, 9234S), antip70S6 (SantaCruz, sc-9202), anti4-EBP1-P (Cell Signaling, 9459S), anti4-EBP1 (Cell Signaling, 9644S), antiGADD34 (SantaCruz, sc-8327), antieiF2*α*-P (Cell Signaling, 9721S), antieiF2*α* (Cell Signaling, 9722S), antiAMPK-P (Cell Signaling, 2531S), antiAMPK (Cell Signaling, 2603S) and antiGAPDH (Santa Cruz, 6C5), and HRP-conjugated secondary antibodies (SantaCruz, sc-2354 and Cell Signaling, 7074S and 7076S). The bands were visualised using chemiluminescence detection kit (Thermo Scientific, 32106).

### 2.4. RNA Interference

RNA interference experiments were performed using Lipofectamine RNAi Max (Invitrogen) in GIBCO™ Opti-MEM I (GlutaMAX™-I) reduced-serum medium liquid (Invitrogen) and 20 pmol/ml siRNA. The siGADD34 oligonucleotides were purchased from ThermoFisher (HSS177543), and the siULK oligonucleotides were purchased from Ambion (AM16708). 200000 HEK293T cells were incubated at 37°C in a CO_2_ incubator in antiobiotic-free medium for 16 hours, and then the RNAi duplex-Lipofectamine™ RNAiMAX (Invitrogen, 13778-075) complexes were added to the cells for overnight. Then fresh medium was added to the cells, and the appropriate treatment was carried out. To check the efficiency of GADD34 silencing, Western blot was used with GADD34 monoclonal antibody (SantaCruz, sc-373815).

### 2.5. RNA Extraction and Real-Time PCR

Total RNA content of cells was extracted using TRIzol RNA isolation reagent (Invitrogen) [33]. Retrotranscription was performed using SuperScriptII First-Strand Synthesis System (Invitrogen). Nucleic acid levels were measured using GenQuant pro RNA/DNA calculator. Equal amounts of cDNA were used for real-time PCR to check the efficiency of GADD34 silencing. PCR reaction and real-time detection were performed using GoTaq(R) qPCR Master Mix (Promega, A6002) and STRATAGENE Mx3005P Real-Time PCR Detection System. The real-time PCR thermocycles were the following: 95°C 10 min (1x), 95°C 30 sec, 58°C 45 sec, 72°C 30 sec (40x), 95°C 5 min, 55°C 1 min, and 97°C 30 sec (1x). The appropriate forward and reverse real-time PCR primers were used for GADD34 and GAPDH.

### 2.6. Cell Viability Assays

The relative amount of viable cells was calculated by Burker chambers. Cell viability was detected using CellTiter-Blue assay (Promega, G8080). Cells were grown and treated on 96-well plates and were incubated with resazurin for 2 h at 37°C. Absorbance was measured at 620 nm and expressed in arbitrary unit, being proportional to cell toxicity. At least three parallel measurements were carried out for each of these experiments.

### 2.7. Statistics

For densitometry analysis, Western blot data were acquired using ImageJ software. The relative band densities were shown and normalized to an appropriate total protein or GAPDH band used as reference protein (see Supplementary Information available here). For each of the experiments, three independent measurements were carried out. Results are presented as mean values ± S.D. and were compared using ANOVA with Tukey's multiple comparison post hoc test. Asterisks indicate statistically significant difference from the appropriate control: ^∗^ *p* < 0.05; ^∗∗^ *p* < 0.01.

## 3. Results

### 3.1. EGCG Affects Autophagy and Apoptosis in a Dose-Dependent Manner

**Figure 1:**
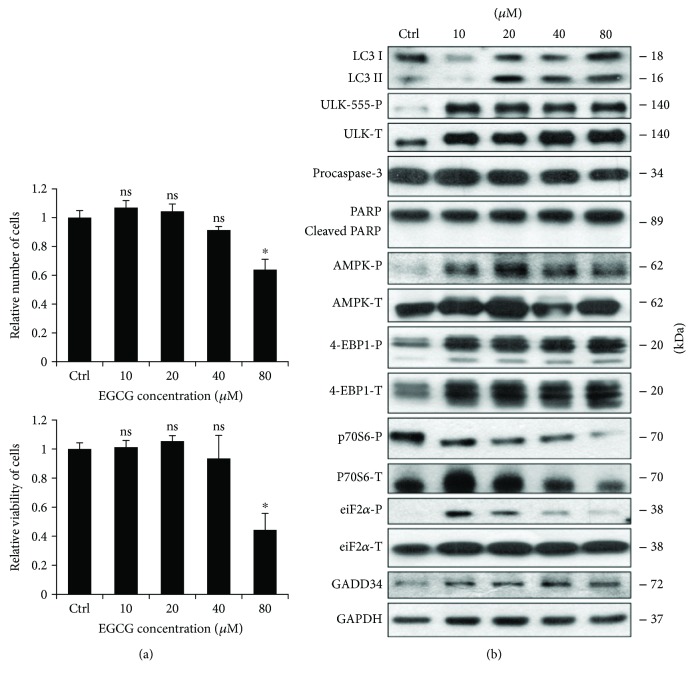
EGCG induces autophagy in a concentration-dependent manner. HEK293T cells were treated with 10, 20, 40, and 80 *μ*M EGCG for 24 h. (a) Meanwhile, the relative number of viable cells (upper panel) and relative cell viability (lower panel) were denoted. (b) During EGCG treatment, the markers of autophagy (LC3, ULK-555-P), apoptosis (procaspase-3, PARP), AMPK (AMPK-P), and mTOR (4-EBP1-P, p70S6-P), as well as ER stress markers (i.e., eiF2*α*-P and GADD34) were followed by immunoblotting. GAPDH was used as loading control. For each of the experiments, three independent measurements were carried out. Error bars represent standard deviation, and asterisks indicate statistically significant difference from the control: ^∗^ *p* < 0.05.

The beneficial health effect of EGCG has been widely studied. It has been shown that low concentrations of EGCG enhance viability of HepG2 cells; however, its high concentration causes a significant decrease in the number of viable cells [34]. Experimental data have also revealed that EGCG is able to enhance both autophagy and apoptosis [35]. In order to figure out whether these cellular processes are correlated to cell viability during EGCG treatment, we further explored the role of green tea polyphenol in cellular decision-making process between life and death. First, human embryonic kidney cells (HEK293T) were treated with various concentrations (10, 20, 40, and 80 *μ*M) of EGCG for 24 h, and we monitored both the relative number of viable cells and cell viability (Figure 1(a)). Corresponding to the already published data, we could confirm that low concentrations of EGCG (i.e., 10 and 20 *μ*M) resulted in a slight increase in cell viability, while excessive level of the polyphenol (i.e., 80 *μ*M) reduced the amount of viable cells by ≈50%.

To detect the activation profile or level of the key indicators of autophagy (such as LC3II and ULK-555-P) and apoptosis (procaspase-3, cleaved PARP) during EGCG treatment, immunoblotting was performed (Figures 1(b) and S1). At low concentration of EGCG (i.e., 10 and 20 *μ*M), a high ratio of LC3II/LC3I and an intensive phosphorylation of ULK-555-P were observed indicating that cell viability is maintained in an autophagy-dependent manner by EGCG in a well-defined concentration range. However, a high concentration of EGCG (80 *μ*M) slightly decreased the activity of autophagy, which was accompanied by a decrease in procaspase-3. Active capase-3 is able to cleave PARP; however, we did not observe any PARP cleavage suggesting that EGCG-dependent apoptosis might occur at a higher concentration of the polyphenol. Interestingly, ER stress was already observed at low concentration of EGCG (see eiF2*α*-P and GADD34 level in Figures 1(b) and S1), although the amount of phosphorylated eiF2*α* was reduced at an excessive level of this natural compound.

It is well-known that EGCG enhances AMPK, but its negative effect on mTOR has not been thoroughly studied yet. To investigate the role of EGCG in modifying the cellular balance of AMPK-mTOR pathways, we detected the key markers of AMPK (AMPK-P, ULK-555-P) and mTOR pathways (such as 4-EBP1-P and p70S6-P) by immunoblotting (Figures 1(b) and S1). EGCG treatment significantly enhances AMPK (see the phosphorylated status of both AMPK and ULK in Figures 1(b) and S1) while mTOR became inactivated. This was detected by both the dephosphorylation of p70S6 and the appearance of the lowest phosphorylation band of 4-EBP1 (Figures 1(b) and S1).

Taken together, these results further confirm that EGCG-induced autophagy is not hazardous for human cells but rather helps maintain cell viability; however, excessive level of this polyphenol might promote an apoptotic cell death. Low dose of EGCG is sufficient to activate AMPK and inhibit mTOR suggesting that EGCG has a key role in unbalancing AMPK-mTOR pathways.

### 3.2. EGCG Induces Autophagy through mTOR-AMPK Pathways

To further explore that EGCG-induced autophagy via unbalancing AMPK-mTOR pathways, we used various drugs to enhance autophagy. It is well known that rapamycin (Rap) treatment induces autophagy via mTOR downregulation [19], while H-89 is a PKA inhibitor and promotes an mTOR-independent autophagy [36] (Figure S2). In order to understand EGCG-induced autophagy, we treated HEK293T cells with either Rap (100 nM, 2 h) or H-89 (2.5 *μ*M, 2 h) and EGCG (20 *μ*M, 24 h) without/with a subsequent Rap (100 nM, 2 h) or H-89 (2.5 *μ*M, 2 h) addition.

**Figure 2:**
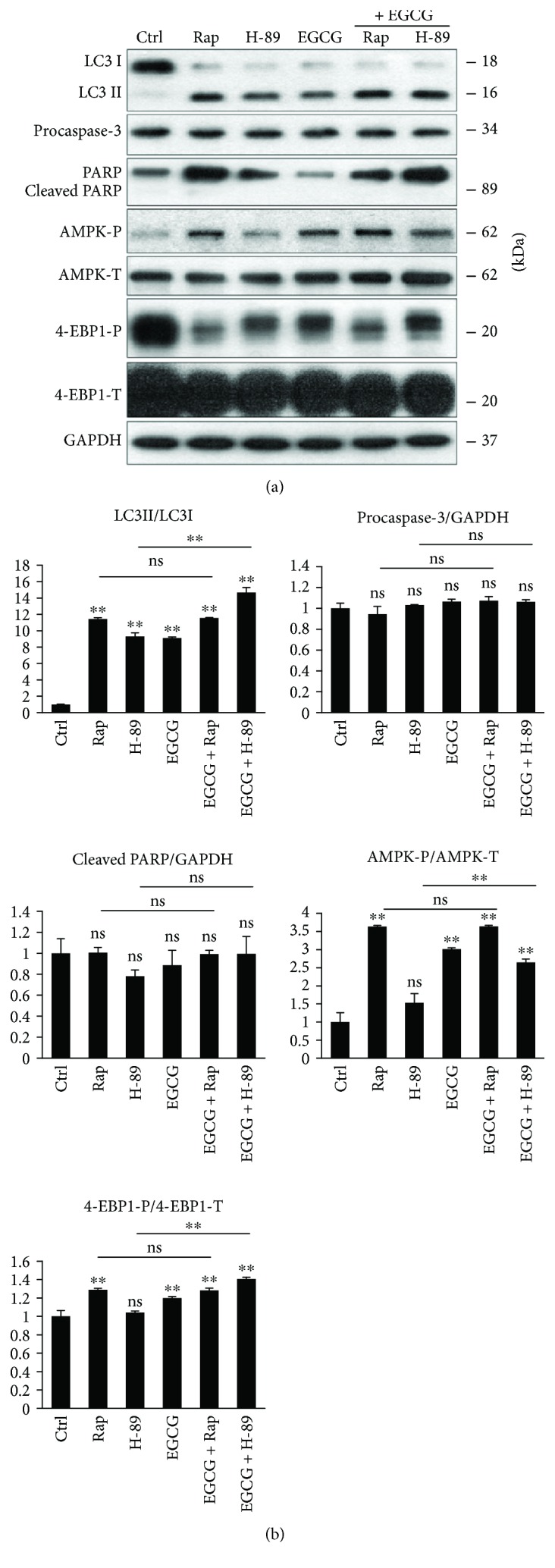
mTOR pathway is essential for EGCG-dependent autophagy induction. HEK293T cells were treated with rapamycin (Rap—100 nM, 2 h), H-89 (2.5 *μ*M, 2 h), and EGCG (20 *μ*M, 24 h) without/with followed by Rap (100 nM, 2 h) or H-89 (2.5 *μ*M, 2 h) addition. (a) The markers of autophagy (LC3), apoptosis (procaspase-3, PARP), AMPK (AMPK-P), and mTOR (4-EBP1-P) were followed by immunoblotting. GAPDH was used as loading control. (b) Densitometry data represent the intensity of procaspase-3, cleaved PARP normalized for GAPDH, LC3II normalized for LC3I, AMPK-P normalized for total level of AMPK, and 4-EBP1-P normalized for total level of 4-EBP1. For each of the experiments, three independent measurements were carried out. Error bars represent standard deviation, and asterisks indicate statistically significant difference from the control: ^∗∗^ *p* < 0.01.

We found that combined treatments (i.e., H-89 + EGCG and Rap + EGCG) did not cause a remarkable decrease in either cell viability or the relative amount of viable cells (Figure S3). Next, the key markers of autophagy, AMPK and mTOR pathways, were detected by immunoblotting (Figure 2). The Rap + EGCG treatment did not cause any additive effect on autophagy induction, AMPK activation, and mTOR downregulation, suggesting that both EGCG and Rap act via the same pathway to induce autophagy. Interestingly, the combined treatment with EGCG and H-89 had a significant additive effect on autophagy induction, indicating that EGCG and H-89 employ different pathways to promote autophagy. Although H-89 itself did not modify the balance of AMPK-mTOR pathways, EGCG was able to activate AMPK (see the phosphorylation of AMPK in Figure 2) and downregulate mTOR (see 4-EBP1-P in Figure 2) in the combined treatment.

Our combinatory treatment experiments suggest that EGCG does not activate autophagy in a PKA-dependent manner. Similarly to Rap, EGCG rather induces autophagy via unbalancing AMPK-mTOR pathways.

### 3.3. ULK1 Is Essential for the EGCG-Induced Autophagy

**Figure 3:**
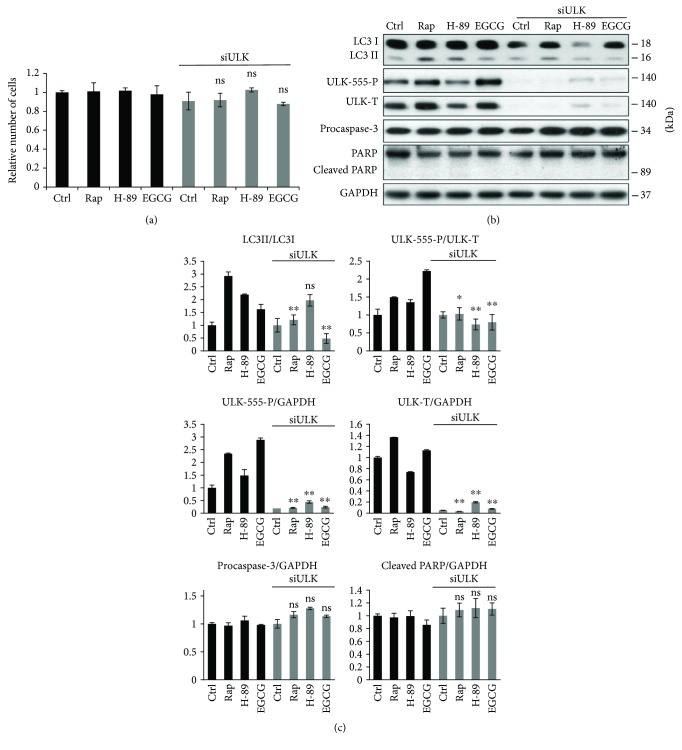
ULK1 is essential for EGCG-dependent autophagy induction. HEK293T cells were treated with rapamycin (Rap—100 nM, 2 h), H-89 (2.5 *μ*M, 2 h), and EGCG (20 *μ*M, 24 h) without/with followed by Rap (100 nM, 2 h) or H-89 (2.5 *μ*M, 2 h) addition. (a) Meanwhile, the relative number of viable cells was denoted. (b) The markers of autophagy (LC3, ULK-555-P) and apoptosis (procaspase-3, PARP) were followed by immunoblotting. GAPDH was used as loading control. (c) Densitometry data represent the intensity of procaspase-3, cleaved PARP, and ULK-555-P and total level of ULK1 normalized for GAPDH, LC3II normalized for LC3I, and ULK-555-P normalized for total level of ULK. For each of the experiments, three independent measurements were carried out. Error bars represent standard deviation, and asterisks indicate statistically significant difference from the control: ^∗^ *p* < 0.05; ^∗∗^ *p* < 0.01.

It is well-known that both AMPK and mTOR regulate autophagy through the phosphorylation of ULK1, one of the key control elements of this cellular process [21]. While AMPK stimulates ULK1 via phosphorylating its Ser-555 and Ser-777, mTOR inhibits autophagy by phosphorylation of different Ser residues in ULK1 (i.e., Ser-757) [21]. Therefore, to further confirm the role of AMPK-mTOR pathways in EGCG-induced autophagy, the effect of EGCG- (20 *μ*M, 24 h) induced autophagy was detected in the presence or absence of ULK1 (Figure 3). We carried out Rap (100 nM, 2 h) and H-89 (2.5 *μ*M, 2 h) treatments for controls. ULK1 knockdown using siULK did not affect the relative amount of viable cells suggesting that ULK depletion did not induce cell death (Figure 3(a)).

Depletion of ULK1 abolished EGCG-induced autophagy (see the low LC3II/I ratio in Figures 3(b) and 3(c)). Similarly to EGCG, Rap was not able to promote autophagy in the absence of ULK1, while siULK did not affect the H-89-induced autophagy (see the LC3II/I ratios in Figures 3(b) and 3(c)). These results further confirm that AMPK-mTOR-regulated autophagy is independent from the PKA pathway.

Taken together, we could conclude that ULK1 is involved in EGCG-induced autophagy and in shifting the balance of mTOR-AMPK pathways.

### 3.4. EGCG Delays Apoptotic Cell Death at an Excessive Level of ER Stress

We have recently identified various drugs (such as metyrapone and resveratrol), which imbalance mTOR-AMPK pathways and thus induce autophagy-dependent survival in ER stress [31, 37]. Since EGCG affects the activation of AMPK and mTOR, we examined whether EGCG also has a positive effect on cell survival during ER stress.

**Figure 4:**
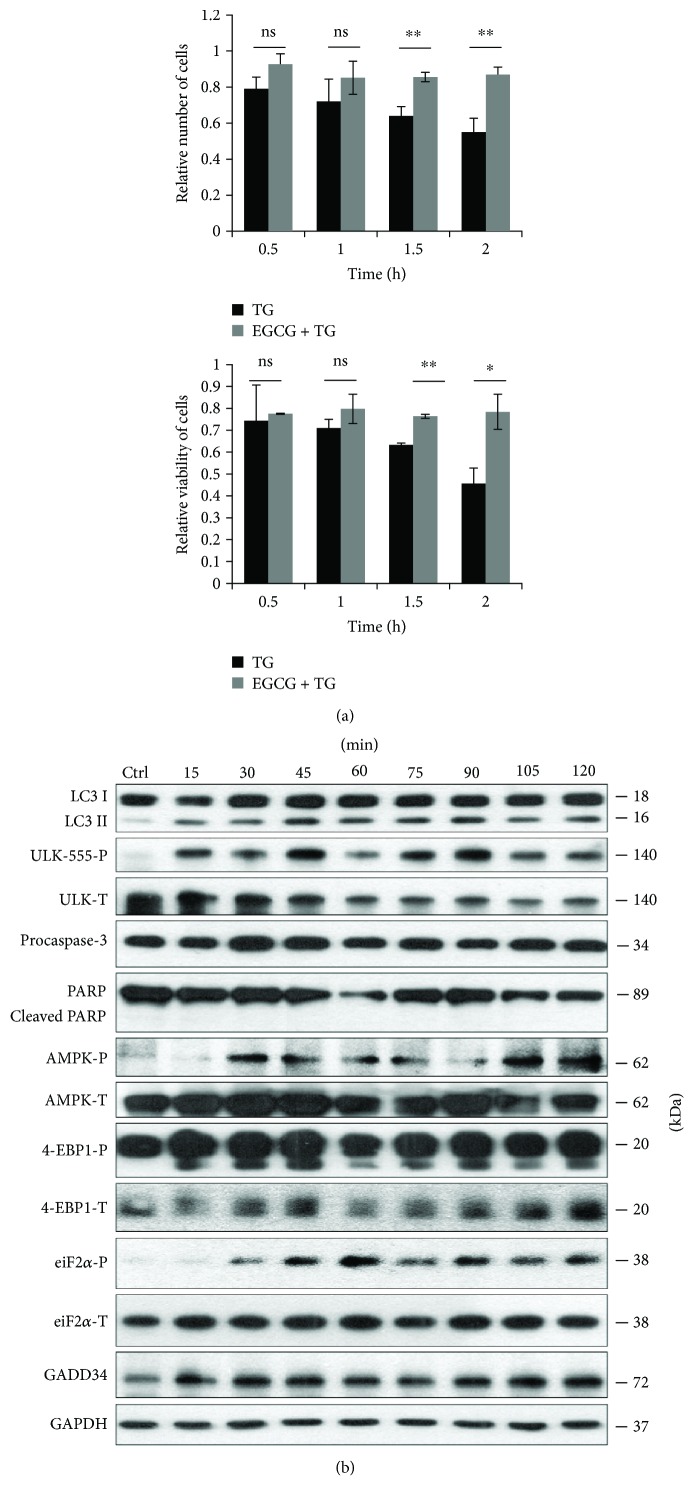
EGCG pretreatment extends autophagy-dependent survival with respect to TG-induced ER stress. HEK293T cells were pretreated with 20 *μ*M EGCG for 24 h followed by TG (10 *μ*M) treatment for 2 h. (a) Meanwhile, the relative number of viable cells (upper panel) and relative cell viability (lower panel) were denoted in time. (b) The markers of autophagy (LC3, ULK-555-P), apoptosis (procaspase-3, PARP), AMPK (AMPK-P), and mTOR (4-EBP1-P), as well as ER stress markers (i.e., eiF2*α*-P and GADD34) were followed by immunoblotting in time. GAPDH was used as loading control. For each of the experiments, three independent measurements were carried out. Error bars represent standard deviation, and asterisks indicate statistically significant difference from the control: ^∗^ *p* < 0.05; ^∗∗^ *p* < 0.01.

**Figure 5:**
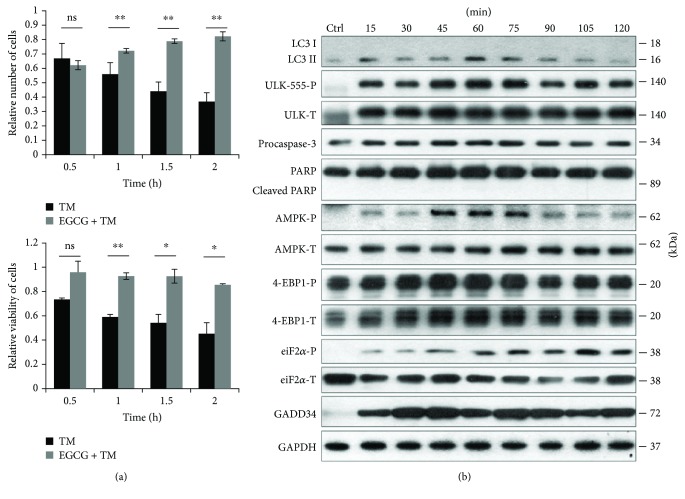
EGCG pretreatment extends autophagy-dependent survival with respect to TM-induced ER stress. HEK293T cells were treated with 20 *μ*M EGCG for 24 h followed by TM (25 *μ*M) treatment for 2 h. (a) Meanwhile, the relative number of viable cells (upper panel) and relative cell viability (lower panel) were denoted in time. (b) The markers of autophagy (LC3, ULK-555-P), apoptosis (procaspase-3, PARP), AMPK (AMPK-P), and mTOR (4-EBP1-P), as well as ER stress markers (i.e., eiF2*α*-P and GADD34) were followed by immunoblotting in time. GAPDH was used as loading control. For each of the experiments, three independent measurements were carried out. Error bars represent standard deviation, and asterisks indicate statistically significant difference from the control: ^∗^ *p* < 0.05; ^∗∗^ *p* < 0.01.

In order to verify the role of EGCG in ER stress, HEK293T cells were pretreated with EGCG (20 *μ*M, 24 h) and then an ER stressor was added, such as thapsigargin (10 *μ*M, 2 h) or tunicamycin (25 *μ*M, 2 h). While thapsigargin (TG) disrupts the calcium storage of the ER, tunicamycin (TM) inhibits N-linked glycosylation of secretory and membrane proteins in the ER [38, 39]. We have already shown that TM- or TG-induced ER stress occurs a transient peak of autophagy-dependent survival followed by apoptotic cell death [29]. To explore whether EGCG is capable to maintain cell viability upon ER stress, both the relative amount of viable cells and relative cell viability were detected during EGCG + TG or EGCG + TM treatments (Figures 4(a) and 5(a)). Addition of EGCG prior to TG or TM significantly extended cell viability and postponed cell death even at continuous treatments with an excessive level of the ER stressor. Our result suggests that this polyphenol is capable of improving cell viability.

In order to detect the effect of EGCG with respect to ER stress, autophagy, apoptosis, AMPK, and mTOR markers were followed during EGCG + TG or EGCG + TM treatments in time by immunoblotting (Figures 4(b), 5(b), S4, and S5). A remarkably high level of LC3II/I suggested that autophagy remained active even after a two-hour-long TG or TM treatment, while neither a drop in procaspase-3 nor the cleavage of PARP was observed. These results indicate that EGCG is able to postpone apoptotic cell death via autophagy induction upon an excessive level of ER stress.

The intensive phosphorylation of both AMPK and ULK (on its Ser-555 residue) suggests that AMPK got stimulated and remained active until the end of the combined treatment (Figures 4(b), 5(b), S4, and S5). The mTOR pathway got downregulated when ER stress was preceded with EGCG addition (see the 4-EBP1-P in Figures 4(b), 5(b), S4, and S5).

These results indicate that EGCG induces autophagy via unbalancing mTOR-AMPK pathways, and by this means it delays apoptotic cell death in ER stress.

### 3.5. Addition of EGCG Can Rescue GADD34 Inhibition with Respect to ER Stress

Recently, we have suggested that one of the key elements of ER stress response mechanism, called GADD34 (the growth arrest and DNA damage-inducible protein) associated with PP1 (protein phosphatase 1), constitutes a mechanistic link between ER stress and mTOR activation [37]. It has been also suggested that GADD34 promotes autophagy-dependent survival via downregulating mTOR in ER stress or in the stress caused by the expression of mutant huntingtin proteins [37, 40]. Inhibition of GADD34 by a PP1 inhibitor (i.e., guanabenz) or transfection with siGADD34 results in a downregulation of autophagy-dependent survival and a quick activation of mTOR pathway, followed by apoptotic cell death during ER stress [37]. Both rapamycin and resveratrol treatments are able to diminish the negative effect of GADD34 downregulation by promoting autophagy induction, AMPK upregulation, and mTOR inhibition [37]. Since EGCG seems to be a potential regulator of mTOR-AMPK balance upon cellular stress, the polyphenol might protect the cells via autophagy induction even in the absence of GADD34 under ER stress.

GADD34 protein level got activated quickly when ER stress was preceded by EGCG addition (Figures 4(b), 5(b), S4, and S5). We observed that GADD34 level remained high even after 2 h long treatment with TM or TG supposing its important role in EGCG-induced autophagy with respect to ER stress.

To explore whether EGCG pretreatment can rescue GADD34 downregulation-induced apoptotic cell death upon ER stress, we carried out a combined treatment. First, HEK293T cells were treated with a GADD34 inhibitor, called guanabenz (GB—5 *μ*M, 1 h), followed by EGCG addition (20 *μ*M) for 24 h. Then ER stress was induced by TG (10 *μ*M, 2 h) or TM (25 *μ*M, 2 h). EGCG pretreatment was able to extend cell viability and increase the relative amount of viable cells in GB-pretreated cells under ER stress (Figure S6).

**Figure 6:**
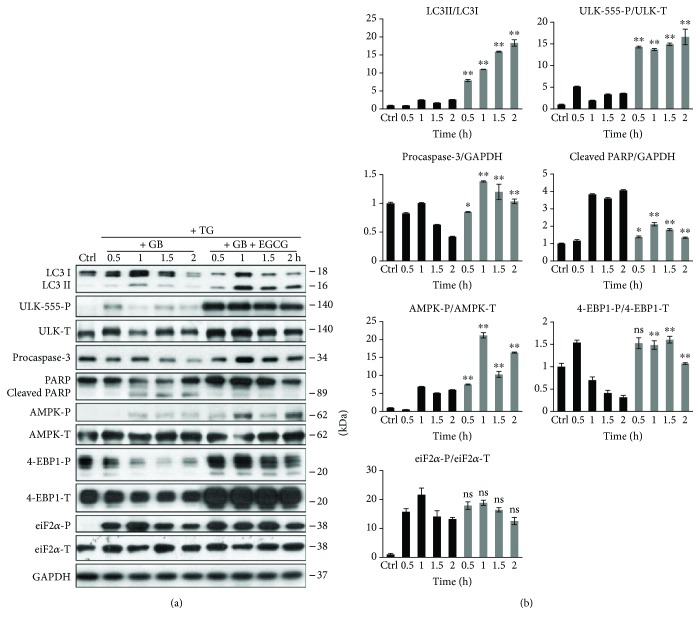
EGCG-dependent effect on mTOR-AMPK pathways rescues GADD34 inhibition with respect to TG-induced ER stress. HEK293T cells were pretreated with GB (5 *μ*M, 1 h) then without/with EGCG (20 *μ*M, 24 h) followed by TG addition (10 *μ*M, 2 h). The GB level was kept high until the end of the cell treatment. (a) After TG treatment, the markers of autophagy (LC3, ULK-555-P), apoptosis (procaspase-3, PARP), AMPK (AMPK-P), and mTOR (4-EBP1-P), as well as ER stress markers (eiF2*α*-P) were followed by immunoblotting. GAPDH was used as loading control. (b) Densitometry data represent the intensity of procaspase-3, cleaved PARP normalized for GAPDH, LC3II normalized for LC3I, ULK-555-P normalized for total level of ULK1, AMPK-P normalized for total level of AMPK, 4-EBP1-P normalized for total level of 4-EBP1, and eiF2*α*-P normalized for total level of eiF2*α*. For each of the experiments, three independent measurements were carried out. Error bars represent standard deviation, and asterisks indicate statistically significant difference from the control: ^∗^ *p* < 0.05; ^∗∗^ *p* < 0.01.

**Figure 7:**
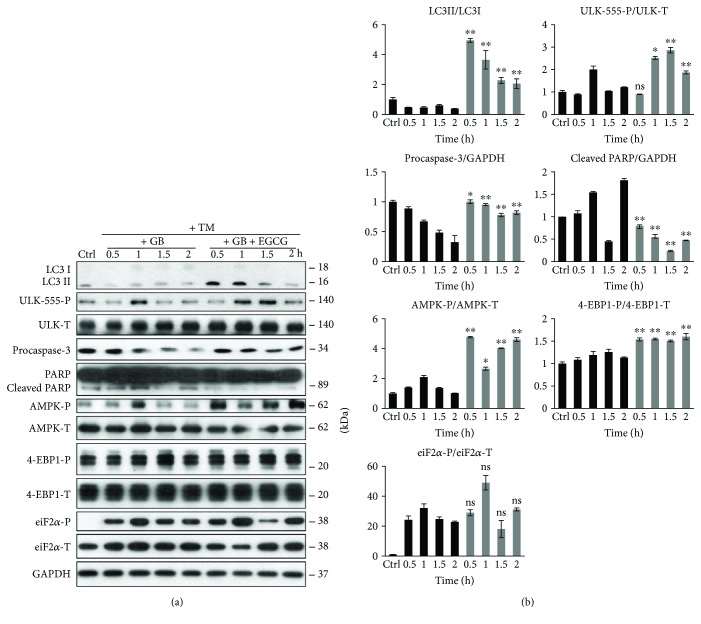
EGCG-dependent effect on mTOR-AMPK pathways rescues GADD34 inhibition with respect to TM-induced ER stress. HEK293T cells were pretreated with GB (5 *μ*M, 1 h) then without/with EGCG (20 *μ*M, 24 h) followed by TM addition (25 *μ*M, 2 h). The GB level was kept high until the end of the cell treatment. (a) After TM treatment, the markers of autophagy (LC3, ULK-555-P), apoptosis (procaspase-3, PARP), AMPK (AMPK-P), and mTOR (4-EBP1-P), as well as ER stress markers (eiF2*α*-P) were followed by immunoblotting. GAPDH was used as loading control. (b) Densitometry data represent the intensity of procaspase-3, cleaved PARP normalized for GAPDH, LC3II normalized for LC3I, ULK-555-P normalized for total level of ULK1, AMPK-P normalized for total level of AMPK, 4-EBP1-P normalized for total level of 4-EBP1, and eiF2*α*-P normalized for total level of eiF2*α*. For each of the experiments, three independent measurements were carried out. Error bars represent standard deviation, and asterisks indicate statistically significant difference from the control: ^∗^ *p* < 0.05; ^∗∗^ *p* < 0.01.

We analysed the effect of GADD34 inhibition during ER stress combined with/without EGCG addition via detecting autophagy, apoptosis, AMPK, and mTOR markers by immunoblotting (Figures 6 and 7). The inactivation of GADD34 was detected by eiF2*α*-P. In the absence of EGCG, GB quickly downregulates autophagy and induces apoptotic cell death during ER stress. However, when cells were pretreated with EGCG, the high ratio of LC3II/I indicates an intensive autophagy until the end of the treatment; meanwhile, apoptosis remains inactive. Neither procaspase-3 depletion nor PARP cleavage was detected in the presence of EGCG. Interestingly, EGCG was able to induce AMPK (see the intensive phosphorylation of both AMPK and ULK1 in Figures 6 and 7) and downregulate mTOR (see 4-EBP1-P in Figures 6 and 7) even if GADD34 was inhibited by GB during ER stress.

These data suggest that EGCG is able to maintain cell viability via autophagy-dependent survival even in the absence of GADD34 upon ER stress. Our experiments indicate that the negative effect of GADD34 inhibition by GB can be suppressed by EGCG-induced imbalance of mTOR-AMPK pathways with respect to ER stress.

### 3.6. GADD34 Silencing by siRNA Has Similar Effects to GB Treatment with Respect to ER Stress

**Figure 8:**
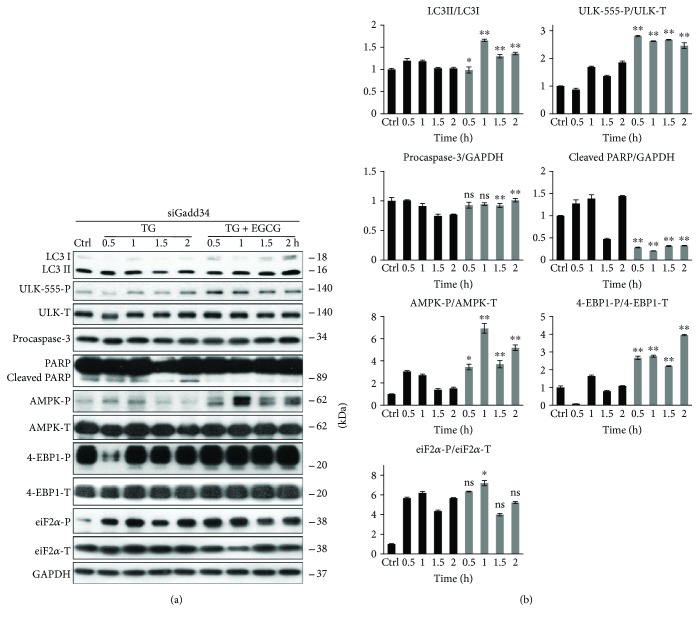
EGCG-dependent effect on mTOR-AMPK pathways rescues GADD34 depletion with respect to ER stress. GADD34 was silenced in HEK293T cells, and then cells were treated with 10 *μ*M TG for 2 h or pretreated with EGCG (20 *μ*M, 24 h) followed by TG addition (10 *μ*M, 2 h). (a) After TG treatment, the markers of autophagy (LC3, ULK-555-P), apoptosis (procaspase-3, PARP), AMPK (AMPK-P), and mTOR (4-EBP1-P), as well as ER stress markers (eiF2*α*-P) were followed by immunoblotting. GAPDH was used as loading control. (b) Densitometry data represent the intensity of procaspase-3, cleaved PARP normalized for GAPDH, LC3II normalized for LC3I, ULK555-P normalized for total level of ULK1, AMPK-P normalized for total level of AMPK, 4-EBP1-P normalized for total level of 4-EBP1, and eiF2*α*-P normalized for total level of eiF2*α*. For each of the experiments, three independent measurements were carried out. Error bars represent standard deviation, and asterisks indicate statistically significant difference from the control: ^∗^ *p* < 0.05; ^∗∗^ *p* < 0.01.

To confirm that EGCG postpones ER stress-induced apoptotic cell death via GADD34, the combined treatment of ER stressor and EGCG was done in cells where GADD34 was silenced with siRNA (Figures 8 and S7). First, we tested the efficiency of siGADD34 both on mRNA (data not shown) and protein (Figure S7A) levels. Similar to addition of GB, GADD34 silencing drastically decreased the amount of viable cells during TG treatment, while pretreatment with 20 *μ*M EGCG for 24 h was able to maintain cell viability (Figure S7B). Addition of TG in HEK293T cells expressing siGADD34 resulted in a short and dumped autophagic response (see the weak LC3II/I ratio and ULK1 phosphorylation in Figure 8), while an early apoptosis induction was observed, that is, depletion of procaspase-3 and appearance of cleaved PARP were already detected after 1.5 h long TG treatment. By contrast, EGCG pretreatment could maintain autophagy-dependent survival and delay apoptotic cell death even in the absence of GADD34 (Figure 8). Both LC3II/LC3I and ULK-555-P levels remain high; meanwhile, no caspase-3 activation was noticed. In these combined treatments, the AMPK also maintained its active state (see the constant phosphorylation of both AMPK and ULK1 in Figure 8), while the mTOR pathway remained blocked (see 4-EBP1-P in Figure 8). Similar effects were observed by using TM (data not shown).

These data further confirm that the negative effect of GADD34 silencing during ER stress can be rescued by EGCG addition. This natural compound is able to imbalance the AMPK-mTOR pathways and promote autophagy-dependent survival in the absence of GADD34.

## 4. Discussion

ER has a key function to maintain cellular homeostasis by containing some of the main regulatory elements of life-and-death decision. Consequently, ER stress-induced damages appear in lots of different human pathologies such as neurodegenerative diseases, obesity, type two diabetes, and many others [41–43]. Using both molecular and theoretical biological techniques, we have shown previously that apoptotic cell death is always preceded by autophagy-dependent survival upon excessive level of ER stress [29, 31]. Therefore, newly identified autophagy inducers might become potent drugs in the future by postponing the injurious effects of ER stress. We have recently confirmed that the “survival window” of autophagy can be expanded by pretreatment with mTOR inhibitors and/or AMPK activators (such as metyrapone and resveratrol) upon ER stress [31, 37]. Here, we introduce a new candidate for extending cell viability, namely, epigallocatechin-3-gallate (EGCG). Plant polyphenols, including green tea flavanols, have pleiotropic effects; however, many of their specific molecular targets have been recently identified. Flavanols are widely known as antioxidants, but under certain conditions (e.g., in the presence of ferric iron) behave as prooxidants [44]. Since they act mainly on cellular membranes, green tea flavanols are known to modulate various functions of the ER [45], including luminal enzyme activities [46, 47], membrane transport processes [48, 49], and redox homeostasis [47]. It has been also demonstrated that EGCG extends life expectancies significantly, which was attributed either to decreased oxidative stress and inflammation [10] or to the induced production of reactive oxygen species [50]. However, the involvement of the AMPK/SIRT1/FOXO axis seems to be firmly established.

Our data demonstrate that a low concentration of EGCG is able to induce autophagy (Figures 1(b) and S1) concomitantly with rise in cell viability, suggesting this activation of self-eating process induced by the polyphenol is not harmful for the cells (Figure 1(a)). Pretreatment with low concentration of EGCG followed by addition of ER stressor (TG or TM) could extend autophagy-dependent survival (Figures 4(b), 5(b), S4, and S5); meanwhile, cell viability did not change (Figures 4(a) and 5(a)) and apoptosis (e.g., PARP cleavage) was not observed upon ER stress (Figures 4(b), 5(b), S4, and S5). Interestingly, Ahn et al. have indicated that the cytotoxic effect of excessive level of EGCG is due to the expression of ER stress response proteins, such as CHOP, GADD34, and ATF3 [34]. Here, we show that the translational initiation factor, eiF2*α*, gets phosphorylated even at low level of EGCG (Figures 1(b) and S1). Although eiF2*α*-P has a key role in shutting down the global protein translation upon ER stress, no cell death is observed suggesting that activation of ER stress response mechanism is not fatal. Rather this eiF2*α* phosphorylation induced by EGCG is essential to upregulate GADD34 level. In this study, we assume that GADD34 level is increased parallel to autophagy induction upon EGCG treatment (Figures 1(b) and S1), indicative of its important role in green tea polyphenol-induced cell survival.

Previously, we have shown that mTOR is downregulated with response to ER stress via GADD34 [37]. We have recently confirmed that blocking GADD34 results in a quick activation of both mTOR pathway and apoptotic cell death; meanwhile, AMPK gets downregulated and the period of autophagy-dependent survival is much shorter upon ER stress [37]. We also supposed that the negative effect of GADD34 depletion is successfully suppressed with mTOR inhibitors and/or AMPK activators (such as rapamycin and resveratrol) during ER stress [37]. To further confirm the role of EGCG in unbalancing mTOR-AMPK pathways, a pharmacological inhibitor (GB) or an siRNA was used to block GADD34 and then cells were pretreated with EGCG followed by addition of an ER stressor. In this study, we show that a 24-hour long pretreatment with a low concentration of green tea polyphenol followed by TG or TM addition was able to extend cell viability via intensive activation of both AMPK and autophagy; meanwhile, mTOR and ER stressor-induced apoptotic cell death were downregulated (Figures 6, 7, and 8). Here, we suggest that EGCG treatment successfully modifies the balance of mTOR-AMPK pathways and thus the negative effect of GADD34 depletion was effectively suppressed. These results further confirm that EGCG-dependent fine-tuning of mTOR-APMK pathways has a crucial effect to maintain the precise balance of life-and-death decision under ER stress.

Since the effect of EGCG on mTOR pathway seems to be contradictory in the literature, EGCG treatment was combined with either mTOR-dependent (rapamycin) or PKA-dependent (H-89) autophagy promoter to identify which pathway is involved in autophagy induction in case of EGCG addition (Figure 2). Autophagy got similarly enhanced both in EGCG and EGCG + Rap treatments revealing that EGCG and Rap regulate the self-eating process via the same mTOR pathway. However, EGCG combined with H-89 significantly promoted autophagy compared to simple H-89 treatment (Figure 2), suggesting that EGCG-induced autophagy is not PKA-dependent. Similarly to rapamycin treatment, transfection with siULK drastically inhibited autophagy during EGCG treatment (Figure 3) confirming that green tea polyphenol induces the self-eating process through ULK1-AMPK-mTOR regulatory network. Since AMPK downregulates mTOR pathway via direct phosphorylation, we cannot rule out that EGCG has both direct and indirect (through AMPK) negative effects on mTOR. Therefore, further studies are needed to identify the exact targets of EGCG.

In conclusion, the positive effects with pretreatment of precisely chosen concentration of EGCG in a human cell line are achieved via promoting autophagy-dependent survival. Therefore, green tea consumption or use of EGCG-loaded nanoparticles or capsules might have therapeutic role in the near future not only in the amelioration of the patients' symptoms suffering from ER stress-related diseases, and in the regulation of body weight as caloric restriction mimetic, but also—obviously not independently from the former effects—to expand lifespan of people. Our interesting findings highlight the potential of EGCG to extend life expectancy by unbalancing mTOR-AMPK pathways via GADD34 upon ER stress.

## Abbreviations

EGCG: Epigallocatechin-3-gallate
mTOR: Mammalian target of rapamycin
AMPK: 5′ AMP-activated protein kinase
Rap: Rapamycin
GB: Guanabenz.

## Supplementary Materials

Supplementary Materials Figure S1: EGCG induces autophagy in a concentration-dependent manner. Figure S2: the effect of H-89 treatment on cell viability. Figure S3: mTOR pathway is essential for EGCG-dependent autophagy induction. Figure S4: EGCG pretreatment extends autophagy-dependent survival with respect to TG-induced ER stress. Figure S5: EGCG pretreatment extends autophagy-dependent survival with respect to TM-induced ER stress. Figure S6: EGCG-dependent imbalance of mTOR/AMPK rescues GADD34 inhibition with respect to ER stress. Figure S7: EGCG-dependent imbalance of mTOR/AMPK rescues GADD34 depletion with respect to ER stress.
