# Supplementary material for: Epigallocatechin-3-Gallate (EGCG) Promotes Autophagy-Dependent Survival via Influencing the Balance of mTOR-AMPK Pathways upon Endoplasmic Reticulum Stress
Source: Oxid Med Cell Longev. 2018 Jan 31;2018:6721530. doi: 10.1155/2018/6721530 (PMC5831959; doi:10.1155/2018/6721530)
Supplement: Supplementary Materials — Figure S1: EGCG induces autophagy in a concentration-dependent manner. Figure S2: the effect of H-89 treatment on cell viability. Figure S3: mTOR pathway is essential for EGCG-dependent autophagy induction. Figure S4: EGCG pretreatment extends autophagy-dependent survival with respect to TG-induced ER stress. Figure S5: EGCG pretreatment extends autophagy-dependent survival with respect to TM-induced ER stress. Figure S6: EGCG-dependent imbalance of mTOR/AMPK rescues GADD34 inhibition with respect to ER stress. Figure S7: EGCG-dependent imbalance of mTOR/AMPK rescues GADD34 depletion with respect to ER stress. [file 6721530.f1.pdf]

Supporting Information

"Epigallocatechin-3-gallate (EGCG) promotes autophagy-dependent survival via influencing the balance of mTOR-AMPK pathways upon endoplasmic reticulum stress"

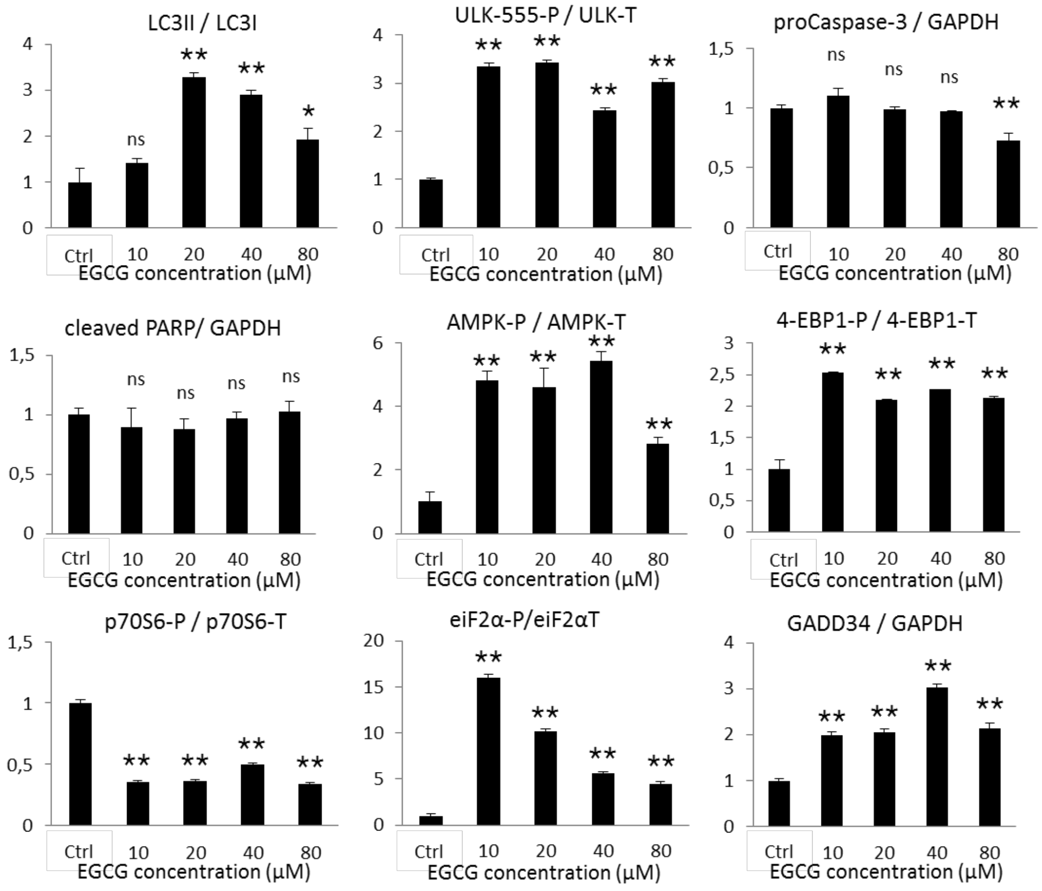

Figure S1. EGCG induces autophagy in a concentration-dependent manner. HEK293T cells were treated with 10, 20, 40 and 80 μM EGCG for twenty-four hours. Densitometry data represent the intensity of proCaspase-3, cleaved PARP, GADD34 normalised for GAPDH, LC3II normalized for LC3I, ULK-555-P normalized for total level of ULK, AMPK-P normalized for total level of AMPK, 4-EBP1-P normalized for total level of 4-EBP1, p70S6-P normalized for total level of p70S6 and eiF2α-P normalized for total level of eiF2α. For each of the experiments, three independent measurements were carried out. Error bars represent standard deviation, asterisks indicate statistically significant difference from the control: ★ - $p < 0.05$; ★★ - $p < 0.01$.

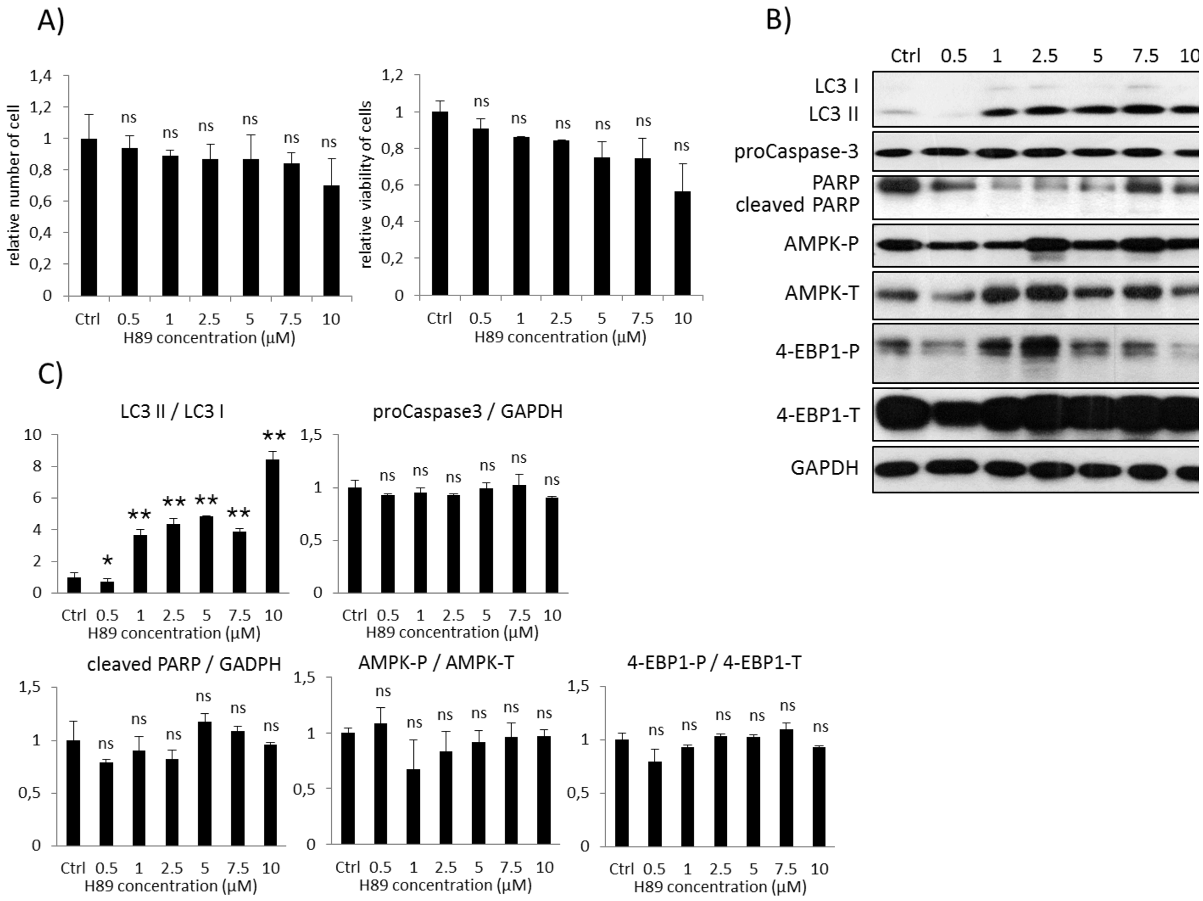

Figure S2. The effect of H-89 treatment on cell viability. HEK293T cells were treated with various concentration of H-89 for two hour meanwhile **A)** the relative number of viable cells **(left panel)** and relative cell viability **(right panel)** were denoted. **B)** During H-89 treatment the marker of autophagy (LC3), apoptosis (proCaspase3, PARP), AMPK activation (AMPK-P) and mTOR activation (4-EBP1-P) were followed by immunoblotting. GAPDH was used as loading control. **C)** Densitometry data represent the intensity of cleaved PARP, proCaspase3 normalised for GAPDH, LC3II normalized for LC3I, AMPK-P normalized for total level of AMPK and 4-EBP1-P normalized for total level of 4-EBP1. For each of the experiments, three independent measurements were carried out. Error bars represent standard deviation, asterisks indicate statistically significant difference from the control: ★ - $p < 0.05$; ★★ - $p < 0.01$.

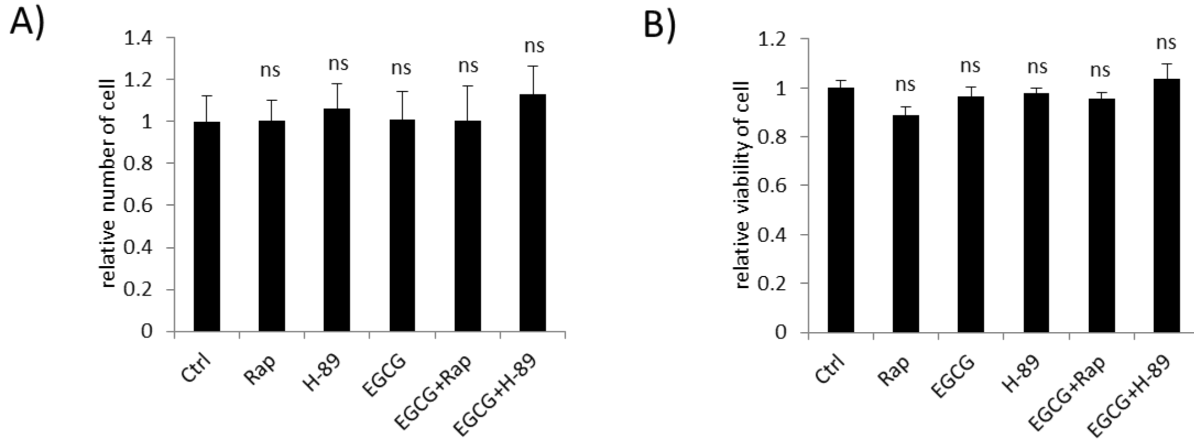

Figure S3. mTOR pathway is essential for EGCG-dependent autophagy induction. HEK293T cells were treated with Rapamycin (Rap – 100 nM, 2 h), H-89 (2.5 μM, 2 h), EGCG (20 μM, 24 h) without/with followed by Rap (100 nM, 2 h) or H-89 addition meanwhile **A)** the relative number of viable cells and **B)** relative cell viability were denoted. For each of the experiments, three independent measurements were carried out. Error bars represent standard deviation, asterisks indicate statistically significant difference from the control: ★ - $p < 0.05$; ★★ - $p < 0.01$.

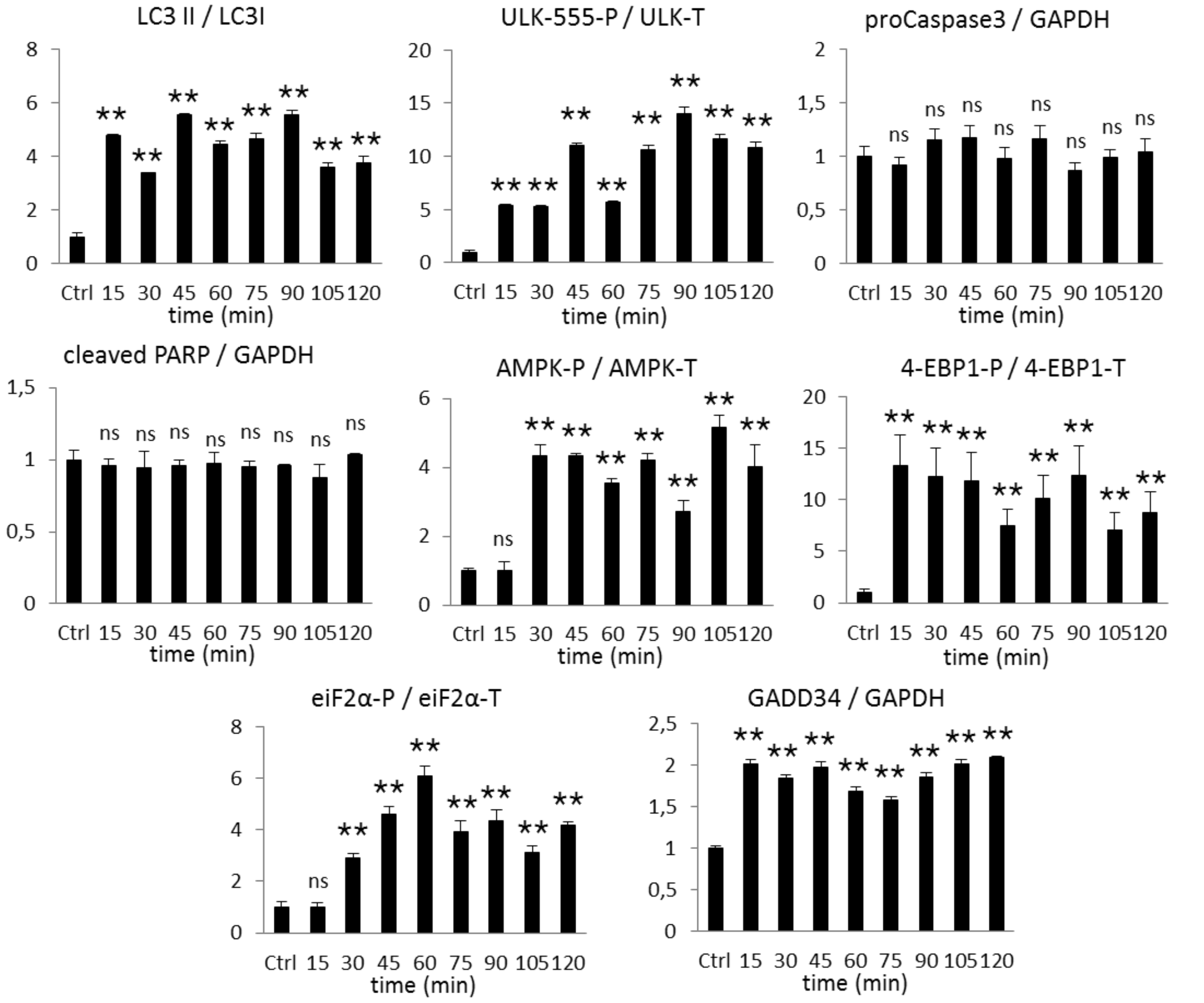

Figure S4. EGCG pre-treatment extends autophagy-dependent survival with respect to TG-induced ER stress. HEK293T cells were treated with 20 μM EGCG for twenty-four hours followed by TG (10 μM) treatment for two hours. Densitometry data represent the intensity of proCaspase-3, cleaved PARP, GADD34 normalised for GAPDH, LC3II normalized for LC3I, ULK-555-P normalized for total level of ULK, AMPK-P normalized for total level of AMPK, 4-EBP1-P normalized for total level of 4-EBP1 and eiF2α-P normalized for total level of eiF2α. For each of the experiments, three independent measurements were carried out. Error bars represent standard deviation, asterisks indicate statistically significant difference from the control: ★ - $p < 0.05$; ★★ - $p < 0.01$.

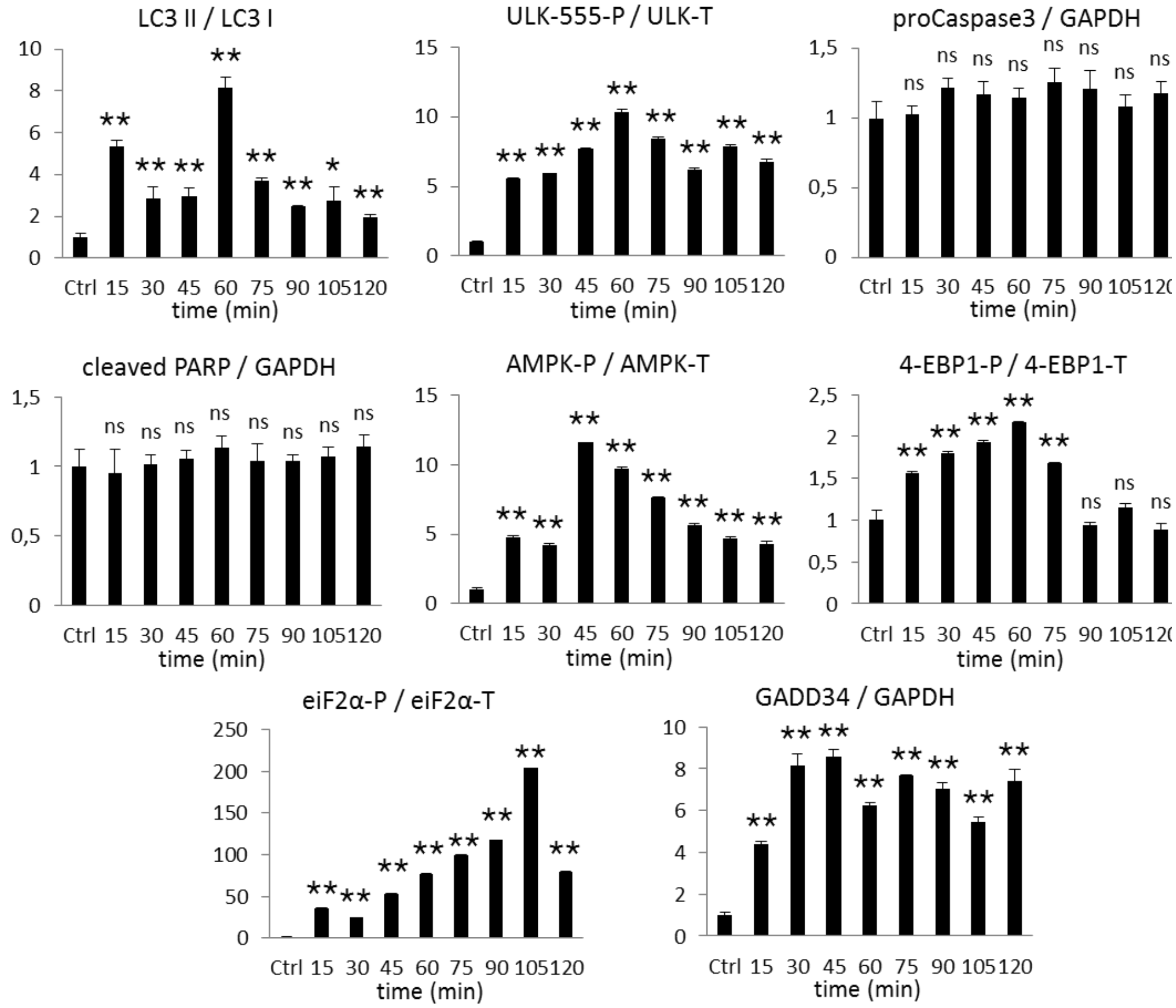

Figure S5. EGCG pre-treatment extends autophagy-dependent survival with respect to TM-induced ER stress. HEK293T cells were treated with 20 μM EGCG for twenty-four hours followed by TM (100 μM) treatment for two hours. Densitometry data represent the intensity of proCaspase-3, cleaved PARP, GADD34 normalised for GAPDH, LC3II normalized for LC3I, ULK-555-P normalized for total level of ULK, AMPK-P normalized for total level of AMPK, 4-EBP1-P normalized for total level of 4-EBP1 and eiF2α-P normalized for total level of eiF2α. For each of the experiments, three independent measurements were carried out. Error bars represent standard deviation, asterisks indicate statistically significant difference from the control: ★ - $p < 0.05$; ★★ - $p < 0.01$.

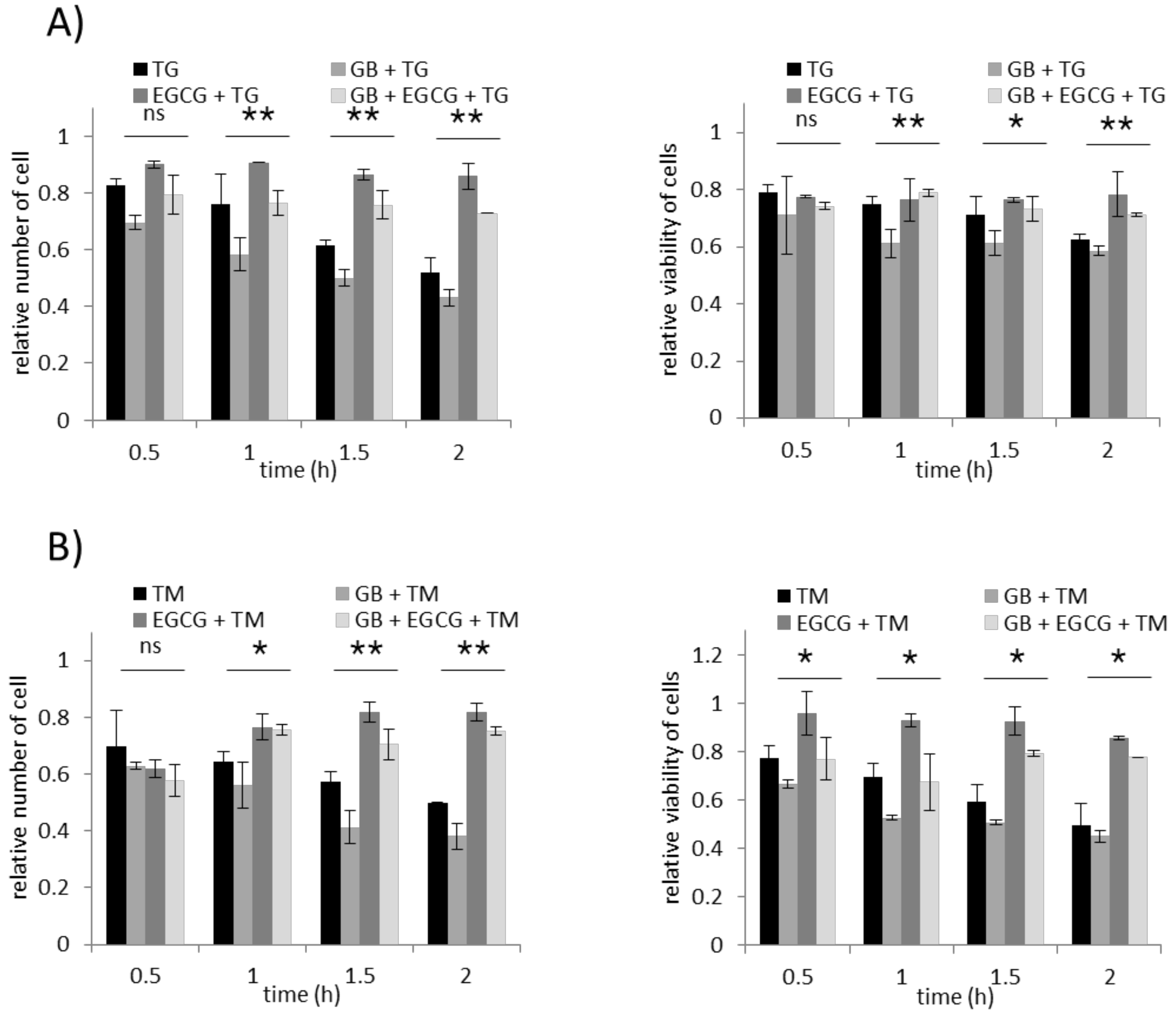

Figure S6. EGCG-dependent imbalance of mTOR/AMPK rescues GADD34 inhibition with respect to ER stress. HEK293T cells were pre-treated with GB (5 µM for one hour) then without/with EGCG (20 µM, 24 hrs) followed by TG (10 µM for two hours – **upper panel**) or TM (25 µM for two hours – **lower panel**) addition meanwhile **A)** the relative number of viable cells and **B)** the relative cell viability were denoted. For each of the experiments, three independent measurements were carried out. Error bars represent standard deviation, asterisks indicate statistically significant difference from the control: ★ - $p < 0.05$; ★★ - $p < 0.01$.

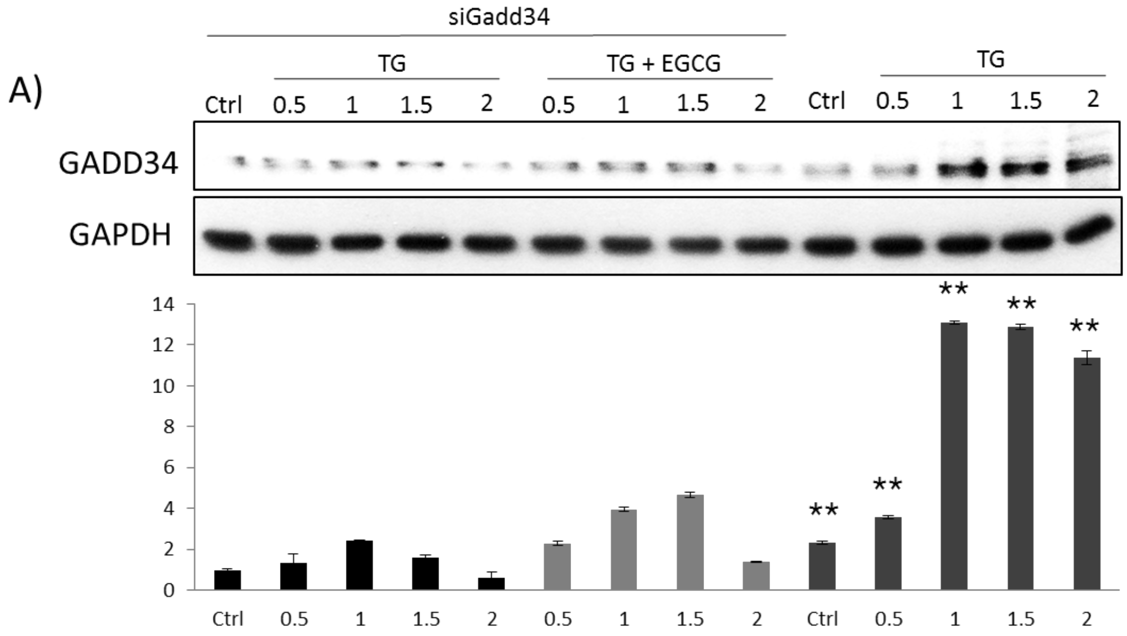

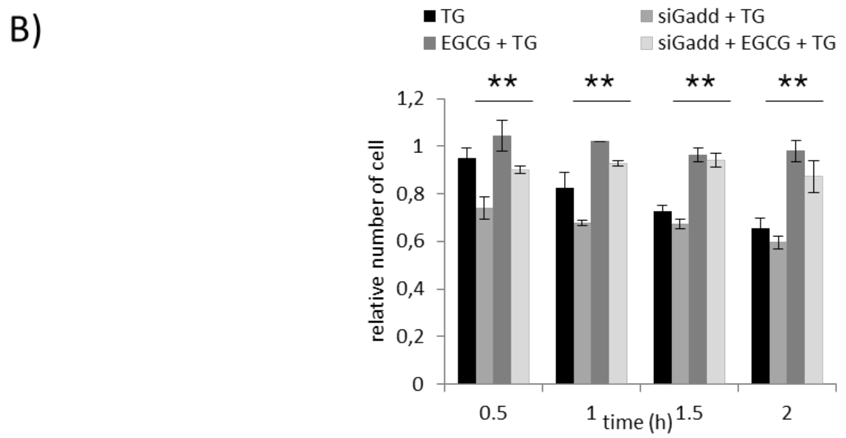

Figure S7. EGCG-dependent imbalance of mTOR/AMPK rescues GADD34 depletion with respect to ER stress. GADD34 was silenced in HEK293T cells, then cells were treated with 10 μM TG for two hours or pre-treated with EGCG (20 μM for twenty-four hours) followed by TG addition (10 μM for two hours). The successful GADD34 silencing was demonstrated by **A)** Western blot analysis and densitometry data represent the intensity of GADD34 normalised for GAPDH. **B)** The relative number of viable cells was denoted in time. For each of the experiments, three independent measurements were carried out. Error bars represent standard deviation, asterisks indicate statistically significant difference from the control: ★ - $p < 0.05$; ★★ - $p < 0.01$.
